# Vaccination Training for Pharmacy Undergraduates as a Compulsory Part of the Curriculum?—A Multicentric Observation

**DOI:** 10.3390/pharmacy12010012

**Published:** 2024-01-11

**Authors:** Shahzad Ahmad Sayyed, Florian Andreas Kinny, Ahmed Reda Sharkas, Holger Schwender, Ronja Woltersdorf, Christoph Ritter, Stephanie Laeer

**Affiliations:** 1 Institute of Clinical Pharmacy and Pharmacotherapy, Heinrich Heine University Duesseldorf, 40225 Duesseldorf, Germany; 2 Mathematical Institute, Heinrich Heine University Duesseldorf, 40225 Duesseldorf, Germany; 3 Institute of Pharmacy, Department of Clinical Pharmacy, University of Bonn, 53121 Bonn, Germany; 4 Institute of Pharmacy, Department of Clinical Pharmacy, University of Greifswald, 17489 Greifswald, Germany

**Keywords:** vaccination training, high-fidelity simulation, pharmacy education

## Abstract

In order to increase vaccination rates, the Government of Germany introduced vaccination against influenza and COVID-19 into the regular care administered by pharmacists. However, vaccination training is yet not integrated into the German pharmacy curriculum. Therefore, the Institute for Clinical Pharmacy and Pharmacotherapy in Duesseldorf had developed an innovative vaccination course using high-fidelity simulation for students. To investigate the acceptance further, the course was carried out at three different German universities (Bonn, Duesseldorf, Greifswald). Students were asked to give their self-assessment before and after and satisfaction only after the training course. Responses from 33 participants from the University of Bonn, 42 from the University of Duesseldorf and 49 from the University of Greifswald were analyzed. Every participant at the respective universities showed a significant increase in their self-assessment and indicated a high level of satisfaction with the course. The results also did not differ significantly between the respective universities. Consequently, the results lead to the hypothesis that the satisfaction of pharmacy students with this kind of training using high-fidelity simulation is very high and attractive, and can be recommended for other German universities. The integration of such vaccination training into the German pharmacy curriculum might be a future step.

## 1. Introduction

Since May 2022, the German government has included vaccination against influenza for persons older than 18 years and against COVID-19 for persons older than 12 years in pharmacies as regular care [1,2]. Under certain conditions, pharmacies are now allowed to offer these vaccinations and be reimbursed by health insurance companies [1,2]. This was preceded by a model project since 2020, in which a few pharmacies had offered the flu vaccination in cooperation with a health insurance company [1]. Furthermore, due to the global COVID-19 pandemic, pharmacists were included in the vaccination campaign in 2021 [3]. These positive experiences finally led to permitting pharmacists to offer vaccinations against these two viruses as regular care under the Infection Protection Act [2].

Vaccination in pharmacies, however, is not a novelty from a global perspective. Pharmacies in many countries offer vaccination services to achieve higher coverage, especially for people older than 65 years of age, for whom a 75% vaccination rate is recommended by the World Health Organization (WHO) [4,5]. In the United Kingdom, pharmacists have been allowed to vaccinate against influenza since 2002 [4]. There, the vaccinations administered by pharmacies for people over 65 years of age increased by 8.15 times between 2015 and 2022, resulting in a vaccination rate of 82.3% in the 2021/22 season [6,7]. In Ireland, where flu vaccination has been allowed in pharmacies since 2011, the service showed that 23% of those who received vaccination at a pharmacy received vaccination for the first time, and of those, 83% were at-risk patients [8]. Norway has also observed an increase in vaccination rates of 32.1% between 2016 and 2020 since the implementation of pharmacy vaccination [9]. Germany reached about 43% vaccination coverage of people over 60 years of age in 2022 [10]. The evidence shows enabling pharmacists to administer vaccines increases vaccination uptake and reaches out to different patient groups compared to conventional means. In Germany, as in other countries such as the USA and Australia, pharmacists are the most accessible healthcare providers [9,11,12,13]. The average density of pharmacies in Germany is 32 pharmacies per 100,000 inhabitants [14]. Here, the services offered play an important role, as well as offering the opportunity for people to speak to a healthcare provider, in this case a pharmacist [15]. This means that more people can be approached and educated about vaccinations [9,11].

Training on vaccination should take place at an early stage in the education of pharmacists. The International Pharmaceutical Federation (FIP) also recommends the incorporation of vaccination training into curricula for pharmacy undergraduates to adequately prepare future pharmacists and to increase the number of vaccinating pharmacists [16]. Furthermore, an overload of the workforce during certain seasons can be reduced by an increased number of vaccinating pharmacists [17]. A report by the FIP in 2020 showed that 35 nations worldwide authorize pharmacy vaccination, of which only 16 offer training for undergraduates, and training is mandatory in just 11 countries [18]. In Germany, pharmacists must complete an additional training course after graduation in order to obtain permission for vaccination in pharmacies [2]. However, in a brief survey by the Federal Chamber of Pharmacists in Germany, only 13% of the participants said they would offer flu vaccination in the future [19]. In order to increase pharmacists’ perception of vaccination at an early stage, vaccination training should be integrated into the curriculum. Therefore, the Institute of Clinical Pharmacy and Pharmacotherapy in Duesseldorf developed a vaccination course for pharmacy students, which uses high-fidelity simulation (HFS) as training tool. The HFS depicts the highest level of realism using simulation. In this case, it is a software-controlled mannequin, allowing us to simulate various vital signs and their changes, such as blood pressure, heart rate or surgical interventions. In comparison, low-fidelity simulation (LFS) is the lowest level of realism offered by simulation, and has no computer-controlled features. For vaccination training, it is a wearable pad with a tissue-like structure [20]. In a randomized controlled study, it was found that training with HFS resulted in better performance among students compared to LFS [21]. Also, students showed increased self-assessment using training with simulators [21]. In this and some other studies, the efficacy of a university course for training pharmacy students on vaccination administration could be demonstrated and evaluated.

In order to investigate whether this specific training on vaccination is not only accepted by the students from Heinrich Heine University Duesseldorf but also by other German pharmacy schools, we expanded our vaccination training course to some other German universities. The primary objective was to ask the students to assess their vaccination performance. Secondly, the satisfaction of the students with the course was also assessed. These two aspects were evaluated by using questionnaires.

## 2. Materials and Methods

### 2.1. Study Design and Participants

In this investigation, the self-assurance and satisfaction of pharmacy students at different universities participating in vaccination training was assessed using a pre- and post-training questionnaire. Four universities were invited to introduce the training course. Of these four universities, two universities agreed to offer the course at their respective universities. In May and June 2023, the vaccination training was given at the universities of Duesseldorf, Bonn and Greifswald. This training was conducted as part of the “clinical pharmacy” course at the respective universities. The responsible faculty members of the respective universities divided the students into groups of 8–12 students and prepared the time schedule. Students were invited to give their consent for the collection of study-related data after receiving detailed participant information. The data were collected pseudonymously and anonymized following analysis. To identify themselves, students should use a code composed of the initials and the last four digits of their student identification number. Approval for this study was granted by the ethics committee of the medical faculty of Heinrich Heine University Duesseldorf (Nr.: 2023-2422).

### 2.2. Training Course

A training course was designed for 8–12 students and lasted 2 h. In the beginning, a short lecture was given to the participants. Thereby, relevant information about the structure and epidemiology of influenza virus and SARS-CoV-2 were given first. Then, the background and global achievements regarding the involvement of pharmacies in vaccination were shown. After this, the content of vaccination training that is required by the regulations was presented, and an introduction to HFS was given. Then, the practical part of the course began, where the participants were asked first to list the requirements for the room and equipment needed for a vaccination in a pharmacy. Second, the important aspects of medical history to determine patient eligibility for vaccination in a pharmacy were listed. In the same way, the aspects of patient education prior to vaccination were stated. The interview for medical history and patient education was then demonstrated by a participant with the HFS. Thereafter, the actions to be taken in an emergency situation and the measurement of patients’ vital signs were explained. At this point, each participant was to perform the vaccination on the simulator, including preparation and injection. During this procedure, five randomly selected participants had to manage an emergency scenario that addressed one of the following cases: anaphylactic reaction, vasovagal syncope, asthma attack, angina attack and hypoglycemic attack. The cases were supervised by a medical doctor and were used in the previous study [21]. Here, the lecturer guided the participants in the proper treatment of the respective scenario. The vaccination and emergency situations were all carried out using the HFS.

### 2.3. Instruments

#### 2.3.1. High-Fidelity Simulator

A high-fidelity simulator (Gaumard HAL^®^S1000; Gaumard Scietific, Miami, FL, USA) was used for the vaccination training and to simulate emergency scenarios. The simulator can be controlled using software, and vital parameters, such as blood pressure, respiratory frequency or pulse, can be changed immediately or after a specified time on the simulator. Participants can also communicate directly with the simulator via a built-in microphone. The injection can be performed in the upper arm. For control during the training sessions, a faculty member sat in a nearby room and answered students’ questions during the scenarios if necessary.

#### 2.3.2. Self-Assessment Questionnaire

To assess the self-assessment of the participants in terms of competency in vaccination in a pharmacy, a self-assessment questionnaire was used, which was developed following the intensive group discussion of faculty members in the previous randomized controlled study [21]. It consisted of six questions with a 6-point Likert scale, where 0 was full disagreement and 5 full agreement. The participants had the possibility to access and complete the questionnaire on a mobile device via QR code. The questionnaire was completed before and after the training course.

#### 2.3.3. Satisfaction Questionnaire

A further questionnaire was designed to evaluate participants’ satisfaction with the vaccination training course. This questionnaire consisted of six questions with a 6-point Likert scale where 0 was full disagreement and 5 full agreement. The questions were related to the use of simulation for clinical practice. Participants also had the possibility to leave a comment or a suggestion for the improvement of the training session. This questionnaire was part of the post-training questionnaire.

### 2.4. Statistical Analysis

In this study, the self-assessment and satisfaction of pharmacy students at multiple universities with the HFS vaccination training was investigated. For the comparison between universities, as well as between pre- and post-training, non-parametric tests were applied. More precisely, to measure the change from the pre-to post-training questionnaire, a Wilcoxon signed-rank test with a significance level of alpha = 0.05 was performed. To determine the difference between the universities in the pre- and post-training questionnaire, the Kruskal–Wallis test with a significance level of alpha = 0.05 was performed. Microsoft Excel 2019 [22] was used for the data entry and OriginPro 2021 [23] for the statistical analysis. The design of the questionnaires and data collection was carried out using the Qualtrics software 2005 [24].

## 3. Results

### 3.1. Participant Characteristics

Ultimately, 130 pharmacy students in the eighth semester participated in the vaccination training and provided informed consent for study-related data collection. Six participants were excluded from the analysis due to missing data and the inability to match pre- and post-training. The participants’ characteristics are described in Table 1. 

### 3.2. Self-Assessment Questionnaire

**Figure 1:**
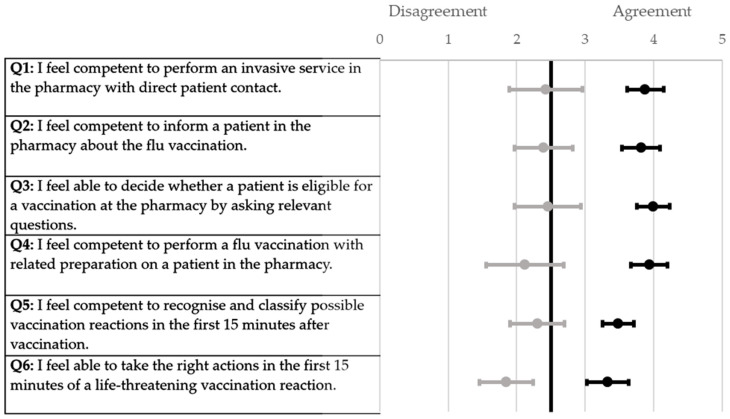
Forest plot of mean values with 95% confidence interval of self-assessment scores pre- and post-training questionnaire (six-point Likert scale) at the University of Bonn. Grey dots (●) = pre-training; black dots (●) = post-training; n = 33.

**Figure 2:**
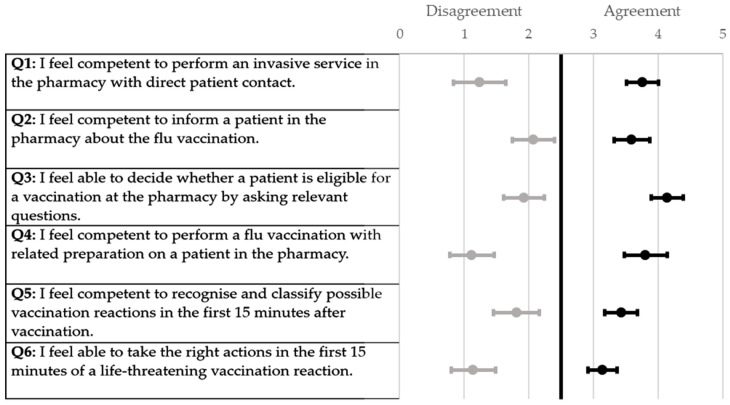
Forest plot of mean values with 95% confidence interval of self-assessment scores pre- and post-training questionnaire (six-point Likert scale) at the University of Duesseldorf. Grey dots (●) = pre-training; black dots (●) = post-training; n = 42.

**Figure 3:**
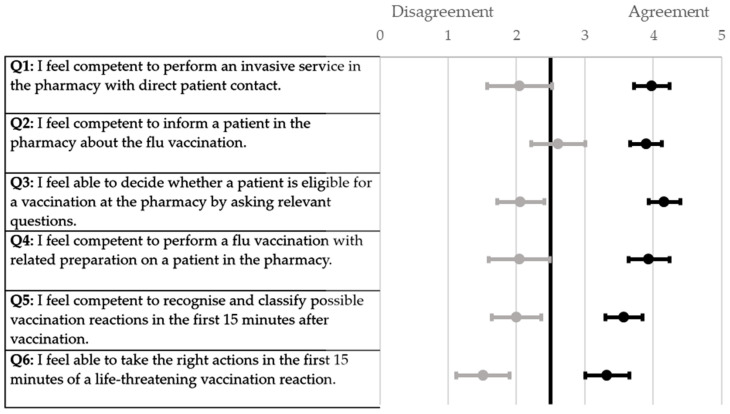
Forest plot of mean values with 95% confidence interval of self-assessment scores pre- and post-training questionnaire (six-point Likert scale) at the University of Greifswald. Grey dots (●) = pre-training; black dots (●) = post-training; n = 49.

**Figure 4:**
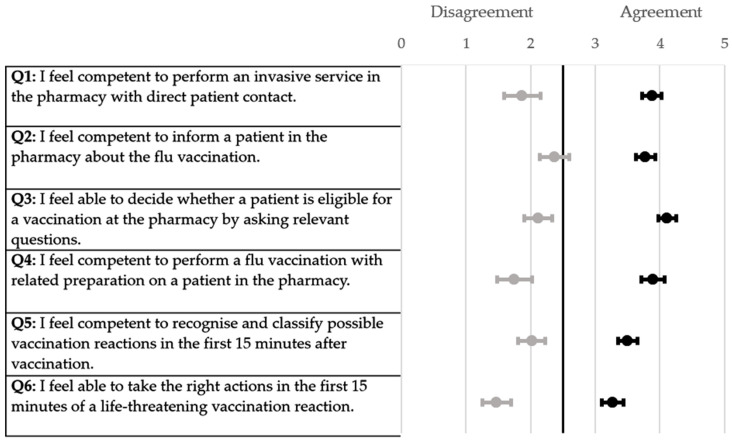
Forest plot of mean values with 95% confidence interval of self-assessment scores pre- and post-training questionnaire (six-point Likert scale) at all universities. Grey dots (●) = pre-training; black dots (●) = post-training; n = 124.

All the participants of the respective universities demonstrated a similar increase in the self-assessment score when ascertained using a six-point Likert scale (Figure 1, Figure 2, Figure 3 and Figure 4). The scores for each question were significantly higher from the pre-to post-training self-assessment questionnaire at each university (Table 2; Figure 1, Figure 2, Figure 3 and Figure 4)). At the University of Bonn, questions 1–5 in the pre-training questionnaire could not be clearly assigned to agreement or disagreement (Figure 1). In contrast, the answers in the pre-training questionnaire at the University of Düsseldorf were assigned firmly to disagreement (Figure 2). At the University of Greifswald, questions 1, 2 and 4 from the pre-training questionnaire could not be assigned clearly to agreement or disagreement (Figure 3). In total, all the results from the pre-training questionnaire are in the range of disagreement, with the exception of question 2 (Figure 4). In the post-training questionnaire, all the results at all three universities were in the range of agreement (Figure 1, Figure 2, Figure 3 and Figure 4). Furthermore, the results do not differ significantly when comparing the universities in both the pre- and post-training self-assessment questionnaires (Table 2). Only in questions 1 and 4 of the pre-training questionnaire were the results significantly different between the three universities, where pairwise comparisons using a Mann–Whitney test showed that the results of the university of Duesseldorf significantly differed from those of the other two (*p*-value < 0.05 in head-to-head comparison of Duesseldorf and other respective universities).

### 3.3. Satisfaction Questionnaire

**Figure 5:**
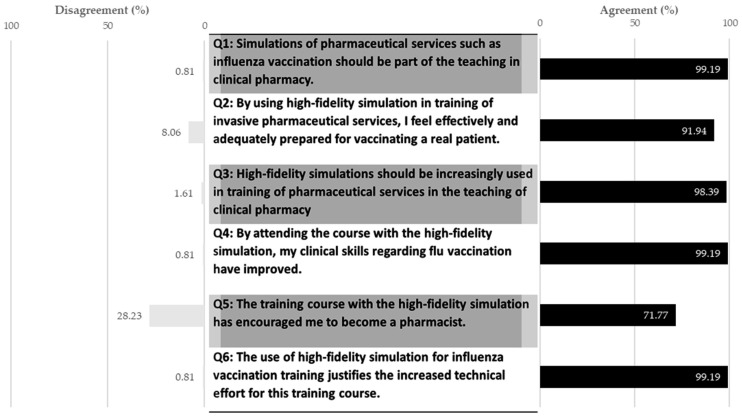
Participants’ satisfaction at all universities in percentage. Agreement indicates positive responses (“strongly agree”, “agree”, “slightly agree”). Disagreement indicates negative responses (“strongly disagree”, “disagree”, “slightly disagree”). n = 124.

Participants declared high satisfaction as rated using a six-point Likert scale. Thereby, the scores of the satisfaction questionnaires did not differ significantly between the universities (Table 3). With the exception of question 5 at the University of Bonn, the scores of all questions were high at all universities (Figure 5). Students described the highest satisfaction in question 1 with the use of simulations in the teaching of clinical pharmacy, whereas the lowest satisfaction was shown in question 5 with the encouragement to become a pharmacist.

## 4. Discussion

In this study, vaccination training using HFS was successfully carried out at three different German universities. It could be demonstrated that the increase in the self-assessment of pharmacy students was significant and similar at all participating universities. At the respective universities, each participant achieved higher self-assessment scores, resulting in a significant increase from pre- to post-training. The participants reported high levels of satisfaction with the training. There were also no significant differences in the results between these universities. Hence, it can be raised that such a vaccination training course could sufficiently prepare students at these universities for vaccination in a pharmacy.

We hypothesize that the integration of a vaccination training course into the pharmacy curriculum could be recommended to these universities. We believe since influenza and COVID-19 vaccination has been included into the standard care offered by pharmacists, such vaccination training during pharmacy school should lead to certification for vaccination practice. In this, as well as in the previous, study, positive outcomes were demonstrated in terms of the student performance and self-assessment at the participating universities [21]. These results line up with the findings of Bushell et al., who also assessed the learning and teaching in vaccination training in pharmacy education in Australia [25]. They observed an increase in knowledge and confidence among students after the training. Mills et al. evaluated the change in attitude, confidence, knowledge and clinical skills among pharmacy students in Australia using pre- and post- questionnaires [26]. They also showed a significant increase in the aforementioned aspects. Marcum et al. assessed the impact of a training program for pharmacy-based immunization for pharmacy students in the USA [27]. The training was found to have a positive impact on students’ knowledge and skills. Another investigation examined the impact of inter-professional training on vaccination administration, where participants from the health disciplines of medicine, nursing and pharmacy received training and ran an immunization clinic [28]. The investigators found a significant increase in knowledge, confidence and skills regarding influenza vaccination. The majority of students who received a vaccination would also recommend it to others. Such an approach shows that using a university training program on vaccination, students are sufficiently qualified to administer vaccinations. Our results underline this with high satisfaction and a significant increase in self-assessment for the students from all participating universities. Also, the comments that the students left in the satisfaction survey after the training were mostly positive. One student wrote “The seminar was very informative and gave a practical insight into the vaccination routine. The concept is very good”. Many other students reported that the training was very helpful and enjoyable. But, from other comments, we can also see that further training sessions are desired, such as in this comment: “More sessions would be helpful. For a more secure feeling, it would be helpful for me to simulate similar emergency situations again at a later date to check that you really recognize situations and take the right measures”.

In this study, HFS was used for the vaccination training, including emergency cases. We believe that by using HFS for vaccination training, besides developing clinical skills, especially the emergency situations that can occur immediately after vaccination can be practiced for effectively. Using the HFS, students can perform inappropriate and unsafe interventions on patients without consequences, and then learn from the effects and further develop their skills and achieve confidence in their interventions using re-attempt and feedback from the instructor [29]. In our studies, the students administered vaccinations and treated emergency scenarios, resulting in increased confidence and satisfaction [21]. Thereby, most students also indicated that HFS should be increasingly used for teaching clinical skills. Numerous studies in various healthcare disciplines have shown an increase in clinical skills among students with the use of HFS [30,31]. More precisely, Jessee et al. showed in an observational study that pharmacy students increased their confidence in applying clinical skills in the field of oncology pharmacy when using HFS training [32]. Morris et al. showed that HFS increases the competence of pharmacists during a medical emergency [33]. We were also able to confirm this in our study, as every student improved in their performance after receiving training with the HFS compared to training with a LFS [21]. However, the effective use of HFS in terms of performance, knowledge and confidence were also shown in other health disciplines and clinical aspects, such as communication, emergency management and inter-professional collaboration [30,34,35,36,37]. 

We are aware that our study is subject to certain limitations. Firstly, the training was only conducted at three universities. The pharmacy schools from the universities recruited via email were not representative concerning the size or regional diversity of Germany. The approach was to ask those universities where some scientific collaboration with the clinical pharmacy department had already been established to increase the chance of collaborating in this scientific approach in the novel vaccination teaching project. To implement such training, the number of students as well as the time schedule during the semester matters. Also, the acquisition of HFS is an expensive undertaking. However, integrating vaccination training into the curriculum is preferable, and using our study, we were able to describe a possible approach to embedding effective vaccination training into the teaching of pharmacy. Future studies should include as many universities as possible in order to make a comprehensive evaluation. Secondly, the duration of the course was limited to 120 min. It was not possible to give a background and general information on influenza, COVID-19 and vaccinations, which would be beneficial to overall knowledge. The training course for pharmacists in Germany and in comparative international settings describes lasts several hours, usually divided into theoretical and practical parts. More precisely, the course for pharmacists in Germany lasts 10.5 h [38]. Similarly, the course offered in Australia includes an 8 h online course and a full-day workshop [39]. However, if vaccination training is integrated into the curriculum, it is necessary to analyze which relevant content needs to be taught in order to obtain certification for vaccination in public pharmacies. In order to ensure a high level of learning during the practical exercise with the HFS, we purposely limited the size of the groups. The number of participants in one group did not exceed 12 [40]. This can also be confirmed by the positive comments of the participants after the course, as they experienced lots of enjoyment and benefited from the course. Theoretical contents should therefore be introduced in a separate lecture. Thirdly, in this study, we did not measure the performance outcomes before and after training. Instead, we collected the participants’ self-assessment before and after the training. We were able to demonstrate a positive effect on performance in the previous study [21]. Also, in terms of the scheduling, time and staffing, it would not be possible to measure performance using objective structured clinical examinations (OSCEs) or other methods. Therefore, during the training sessions, each participant was closely monitored and corrected immediately by the instructor in case of errors. 

## 5. Conclusions

Pharmacists in Germany and globally occupy a special role in patient care, including vaccination. Accordingly, pharmacists must already be adequately and effectively prepared for future challenges during their professional education. Therefore, a vaccination course seems to be highly accepted by the students at these three different locations. It could be integrated into the pharmacy curriculum. Furthermore, students recommended this kind of training also to colleagues. Therefore, other pharmacy schools in Germany might also profit from this kind of vaccination training program using innovative technologies such as high-fidelity simulation.

## Figures and Tables

**Table 1:** Participant characteristics.

	Bonn (n = 33)	Duesseldorf (n = 42)	Greifswald (n = 49)	Total (n = 124)
**Age**				
**Mean (±SD)**	23.91 (±1.88)	24.79 (±3.54)	23.31 (±1.79)	23.97 (±2.60)
**Median**	24	24	23	23
**Range**	21–28	21–38	21–28	21–38
**Gender**				
**Female, n (%)**	23 (69.70)	32 (76.19)	28 (57.14)	83 (66.94)
**Male, n (%)**	10 (30.30)	10 (23.81)	21 (42.86)	41 (33.06
**Previous or current experience (e.g., pharmaceutical technician, vaccination centre)**				
**No, n (%)**	28 (84.85)	35 (83.33)	37 (75.51)	100 (80.65)
**Yes, n (%)**	5 (15.15)	7 (16.67)	12 (24.49)	24 (19.35)

SD = standard deviation.

**Table 2:** Achieved scores by participants in self-assessment questionnaire.

		Bonn (n = 33) Mean (CI)	Duesseldorf (n = 42) Mean (CI)	Greifswald (n = 49) Mean (CI)	Total (n = 124) Mean (CI)	*p*_2_-Value
**Q1**	**Pre-Training**	2.42 (0.53)	1.24 (0.41)	2.04 (0.47)	1.87 (0.28)	<0.01
	**Post-Training**	3.88 (0.27)	3.76 (0.25)	3.98 (0.26)	3.88 (0.15)	0.26
	***p*_1_-Value**	<0.01	<0.01	<0.01	<0.01	
**Q2**	**Pre-Training**	2.39 (0.43)	2.07 (0.33)	2.61 (0.40)	2.37 (0.23)	0.17
	**Post-Training**	3.82 (0.28)	3.60 (0.28)	3.90 (0.23)	3.77 (0.15)	0.31
	***p*_1_-Value**	<0.01	<0.01	<0.01	<0.01	
**Q3**	**Pre-Training**	2.45 (0.48)	1.93 (0.32)	2.06 (0.34)	2,12 (0.22)	0.18
	**Post-Training**	4.00 (0.24)	4.14 (0.25)	4.16 (0.23)	4.11 (0.14)	0.47
	***p*_1_-Value**	<0.01	<0.01	<0.01	<0.01	
**Q4**	**Pre-Training**	2.12 (0.57)	1.12 (0.34)	2.04 (0.45)	1.75 (0.27)	<0.01
	**Post-Training**	3.94 (0.27)	3.81 (0.33)	3.94 (0.30)	3.90 (0.18)	0.74
	***p*_1_-Value**	<0.01	<0.01	<0.01	<0.01	
**Q5**	**Pre-Training**	2.30 (0.40)	1.81 (0.35)	2.00 (0.36)	2.02 (0.21)	0.24
	**Post-Training**	3.48 (0.23)	3.43 (0.25)	3.57 (0.27)	3.50 (0.15)	0.65
	***p*_1_-Value**	<0.01	<0.01	<0.01	<0.01	
**Q6**	**Pre-Training**	1.85 (0.39)	1.14 (0.34)	1.51 (0.39)	1.48 (0.22)	0.04
	**Post-Training**	3.33 0.30)	3.14 (0.23)	3.33 (0.32)	3.27 (0.17)	0.37
	***p*_1_-Value**	<0.01	<0.01	<0.01	<0.01	

CI = 95% confidence interval; *p*_1_-value = intragroup comparison; *p*_2_ = intergroup comparison.

**Table 3:** Achieved scores by participants in satisfaction questionnaire.

	Bonn (n = 33) Mean (CI)	Duesseldorf (n = 42) Mean (CI)	Greifswald (n = 49) Mean (CI)	Total (n = 124) Mean (CI)	*p*-Value
**Q1**	4.55 (0.24)	4.69 (0.18)	4.49 (0.21)	4.57 (0.12)	0.22
**Q2**	3.67 (0.30)	3.69 (0.29)	3.88 (0.25)	3.76 (0.16)	0.60
**Q3**	4.48 (0.27)	4.55 (0.20)	4.43 (0.22)	4.48 (0.13)	0.64
**Q4**	4.39 (0.26)	4.38 (0.23)	4.39 (0.24)	4.39 (0.14)	0.94
**Q5**	2.88 (0.47)	3.10 (0.38)	3.39 (0.41)	3.15 (0.24)	0.25
**Q6**	4.36 (0.25)	4.24 (0.24)	4.39 (0.21)	4.33 (0.13)	0.67

## Data Availability

The dataset presented in this study is available from the corresponding author on reasonable request.
